# Modelling interspecific hybridization with genome exclusion to identify conservation actions: the case of native and invasive *Pelophylax* waterfrogs

**DOI:** 10.1111/eva.12245

**Published:** 2015-01-28

**Authors:** Claudio S Quilodrán, Juan I Montoya-Burgos, Mathias Currat

**Affiliations:** 1 Laboratory of vertebrate evolution, Department of Genetics and Evolution, University of GenevaGeneva, Switzerland; 2 Laboratory of anthropology, genetics and peopling history, Anthropology Unit, Department of Genetics and Evolution, University of GenevaGeneva, Switzerland

**Keywords:** biological invasion, demographic threat, exotic species, hemiclones, hybridization model, hybridogenesis

## Abstract

Interspecific hybridization occurs in nature but can also be caused by human actions. It often leads to infertile or fertile hybrids that exclude one parental genome during gametogenesis, escaping genetic recombination and introgression. The threat that genome-exclusion hybridization might represent on parental species is poorly understood, especially when invasive species are involved. Here, we show how to assess the effects of genome-exclusion hybridization and how to elaborate conservation actions by simulating scenarios using a model of nonintrogressive hybridization. We examine the case of the frog *Pelophylax ridibundus*, introduced in Western Europe, which can hybridize with the native *Pelophylax lessonae* and the pre-existing hybrid *Pelophylax esculentus,* maintained by hybridogenesis. If translocated from Southern Europe, *P. ridibundus* produces new sterile hybrids and we show that it mainly threatens *P. esculentus*. Translocation from Central Europe leads to new fertile hybrids, threatening all native waterfrogs. Local extinction is demographically mediated via wasted reproductive potential or via demographic flow through generations towards *P. ridibundus*. We reveal that enlarging the habitat size of the native *P. lessonae* relative to that of the invader is a promising conservation strategy, avoiding the difficulties of fighting the invader. We finally stress that nonintrogressive hybridization is to be considered in conservation programmes.

## Introduction

Three types of interspecific hybridization can be defined according to the reproductive characteristics of the first generation hybrids: (i) hybridization yielding unviable or infertile offspring; (ii) genome-exclusion hybridization producing F_1_ hybrids that exclude one parental genome during gametogenesis and, therefore avoiding genetic introgression between the parental species; and (iii) hybridization generating F_1_ hybrids in which genetic recombination between the parental genomes proceeds normally during gametogenesis. The threat to parental species that hybridization with genome exclusion may represent is certainly not due to genetic introgression but can be caused by a demographic decline in one or both species, yet this phenomenon is poorly understood. In conservation biology, demography is considered of primary importance in determining the viability of wild populations (Lande 1988). In particular, the viability of small-sized populations depends on the interaction between demographic and genetic factors, both playing a role during a demographic decline. A minimum population size has to be maintained to avoid inbreeding depression in the short term and retaining sufficient genetic variation to allow adaptive changes over large timescales (Jamieson and Allendorf 2012).

In addition to the potential threat that interspecific hybridization between native species may represent, especially in the context of global change, interspecific hybridization may facilitate biological invasions by accelerating the population decline of native species (Hall and Ayres 2009). The invasion of exotic species is one of the main threats to biodiversity worldwide, in which competition, predation or even habitat modification by exotic species can lead native taxa to the brink of extinction (Mack et al. 2000). By modelling interspecific hybridization, it is possible to assess the future consequences on the demography of parental species or hybrid populations and to predict the conditions under which local populations or even a species can reach extinction.

Here, we present an extensive investigation of the possible outcomes on the demography of native species threatened by the introduction of an alien species with which they hybridize, and we project the impact of different changes in the biological setting to identify effective conservation actions. To this aim, we adapted and implemented a model of interspecific hybridization without genetic introgression that we have recently developed (Quilodrán et al. 2014). The case study of biological invasion reinforced by nonintrogressive hybridization that we investigated here is the colonization of Western Europe by the waterfrog *Pelophylax ridibundus* coming either from Central or Southern Europe (Holsbeek and Jooris 2010), apparently mediated by human activities (Luquet et al. 2011). In France and Switzerland, this waterfrog was introduced during the 20th century for frog legs consumption and for scientific purposes. Since then, this exotic species has been displacing local populations of native waterfrogs probably facilitated by the fact that it can hybridize with the native *Pelophylax lessonae* (Vorburger and Reyer 2003). This threat to native frogs adds to the global worldwide threat to amphibians caused by human-induced climate change (Shoo et al. 2011).

**Figure 1:**
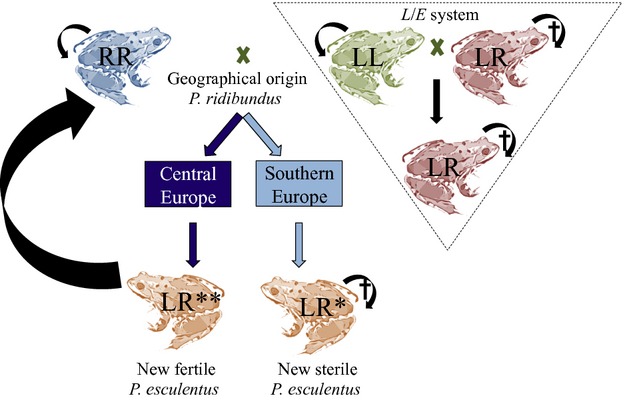
Impact of the exotic *Pelophylax ridibundus* on the waterfrog *L/E* system of Western Europe. Straight and curve arrows represent heterotypic and homotypic matings, respectively. The black cross symbol means that mating leads to unviable offspring. LL = *Pelophylax lessonae*; RR = *P. ridibundus*; LR = *P. esculentus*; LR* = new sterile *P. esculentus*; LR** = new fertile *P. esculentus* (see main text). Dark and sky blue arrows differentiate new fertile from new sterile hybrids, respectively. The hybrids (*P. esculentus* LR) were already present in the invaded area and have been persisting by hybridogenesis with *P. lessonae* (the native *L*/*E* system). Exotic *P. ridibundus* coming from Southern Europe produce new sterile hybrids, whereas when coming from Central Europe, they produce new fertile hybrids, whose offsprings are again *P. ridibundus*.

In nature, hybrids between *P. ridibundus* and *P. lessonae* were present in Western Europe before the recent introduction of *P. ridibundus*; they have been named *Pelophylax esculentus* and have long been considered as a different species. Previous genetic studies revealed the hybrid nature of *P. esculentus*, which likely originated at the time of the last glaciation, about 10 000 years ago, when native populations of *P. ridibundus* were suggested to be present in Western Europe but went subsequently extinct (Vorburger 2001). The hybrid *P. esculentus* persisted by hybridogenesis with *P. lessonae* despite the extinction of *P. ridibundus* (Fig.1). This hybridogenetic system is characterized by the hybrid *P. esculentus*, which develops germ cells that discard the *P. lessonae* genome before meiosis, producing only haploid gametes containing the *P. ridibundus* genome (Anholt et al. 2003). Thus, backcrosses between *P. esculentus* and *P. lessonae* generate only *P. esculentus* hybrids. Crosses between native *P. esculentus* hybrids are unsuccessful likely due to the expression of recessive deleterious mutations (Vorburger and Reyer 2003). This hybridogenetic system has been named the *L*/*E* system (Graf 1986).

The equilibrium of the *L*/*E* system was disrupted in Western Europe by the anthropogenic introduction of *P. ridibundus* from different places of Central and Southern Europe (Schmeller et al. 2007). It has been recently highlighted that populations of *P. ridibundus* from different geographical origins differ in the type of offspring they produce (Holsbeek and Jooris 2010; Plötner et al. 2010). Introduced individuals coming from Southern Europe, when mating with the native *P. lessonae*, produce new *P. esculentus* hybrids that are sterile in all types of crosses (see Fig.1). In contrast, if the parental *P. ridibundus* comes from Central Europe, the resulting *P. esculentus* hybrids are fertile in all types of crosses. The latter situation is explained by the probable absence of recessive deleterious mutations in the genome of *P. ridibundus* coming from Central Europe (Holsbeek and Jooris 2010).

The introduction of *P. ridibundus* in Western Europe is likely to cause complex interactions and perturbations in the native *L*/*E* system, like ecological interspecific competition, waste of reproductive potential for native taxa when they mate with the invasive species or population replacement through hybrid production and asymmetric backcrosses with parental species. Therefore, predicting the risks to resident populations and proposing efficient protection strategies are not trivial tasks. Models that describe the native *L*/*E* system in Western Europe have already been proposed (Graf 1986; Hellriegel and Reyer 2000; Som et al. 2000; Som and Reyer 2006), but our model is more general and adaptable because important processes like density-dependent competition, dominance/recessive inheritance of traits and assortative mating are incorporated. It allows us to include into the system the populations of the invasive *P. ridibundus* coming from different origins, with their reproductive specificities, an element which was absent from previous models. Thus, using our model as a tool to simulate different scenarios of invasion and to assess their consequences in the biological system, we aimed at: i) generating a comprehensive picture of the threats that native frogs may face, ii) determining the conditions under which native waterfrogs may be at risk, and iii) identifying key factors that can be modified for deploying an efficient conservation strategy.

## Materials and methods

Our aim was to describe and assess the possible outcomes of the invasion of Western Europe by different populations of *P. ridibundus* (*R*) showing different reproductive properties when they hybridize with native *P. lessonae* (*L*) and with the previously present hybrid *P. esculentus* (*E*). For this aim, we adapted (see Appendix S1 for details) the model of distant species hybridization without genetic introgression between parental species that we have recently developed (Quilodrán et al. 2014). In our simulations, to assure that native frogs reach a stable equilibrium before the invasion of *P. ridibundus,* we let the native *L*/*E* system evolve for 200 time steps (years). For the invasion of the exotic *P. ridibundus* populations, and because we do not know the exact initial number of introduced frogs, we simulated a translocation of *P. ridibundus* representing 0.2% of the total frog community, emulating the invasion from a small population source as it is the case for most species translocated by humans (Fitzpatrick et al. 2012). We explored the behaviour of the simulations in changing this initial population size from 0.02% to 2%, yet the results and conclusions of our model remained similar (data not shown). In the model, we used *N*_*L*_, *N*_*E*_, *N*_*R*_ to refer to adult frog population sizes (*L* for *P. lessonae*,*E* for *P. esculentus* and *R* for *P. ridibundus*).

### The *L*/*E* system

In the native *L*/*E* system, the homotypic mating of *P. esculentus* (*E *× *E*) does not produce viable offspring (Fig.1). Therefore, populations of *P. lessonae* and *P. esculentus* depend exclusively on the homotypic cross *L *× *L* and on the heterotypic cross *E *× *L*, respectively. Assuming equal sex ratio, we used eqn 1 to calculate the weighted number of breeding events leading to offspring of type *L* (*n*_*L*_), based on eqn 3 in Quilodrán et al. (2014):

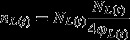
1 where *φ*_*L*(*t*)_ is a normalization factor such that the proportion of homotypic and heterotypic mating of *L* is equal to 1 (Appendix S1).

The heterotypic cross between *P. esculentus* and *P. lessonae* produces hybrid offspring with 1:1 sex ratio if the male is *P. lessonae* (*E*^*f*^* *× *L*^*m*^), or only female hybrid offspring if the male is *P. esculentus* (*L*^*f*^ × *E*^*m*^). This is because *P. esculentus* (both males and females) produces gametes containing always the *R* genome (*P. ridibundus*) with the X chromosome (Berger 1988; Berger et al. 1988). Sex determination in these frogs is XX-XY (males being heterogametic). To account for the unequal sex ratio in the offspring type *P. esculentus,* we calculate separately the number of breeding events involving males (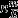) and females (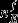), using the following equations:

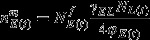
2

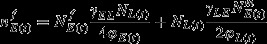
3

The parameter *γ* represents the interbreeding success rate, as introduced in Currat et al. (2008) and Quilodrán et al. (2014). When *γ* is equal to zero, there is a complete reproductive isolation whereas a value of 1 corresponds to a panmictic reproduction between both species. With any other value of *γ ∈* [0, 1], the mating occurs more often between members of the same species. Here, *γ*_*EL*_ and *γ*_*LE*_ are the interbreeding success rate between females *E* and males *L* (*E*^*f*^ × *L*^*m*^) and between females *L* and males *E* (*L*^*f*^ × *E*^*m*^), respectively.

### The newly formed hybrids

To track the reproductive consequences of the introduced *P. ridibundus* depending on their geographical origin, the population dynamics of new sterile E^*^ and new fertile hybrids E^**^ were differentiated from that of the old hybrids *P. esculentus E*. For the new hybrids, we assume an equal sex ratio, because E^*^ and E^**^ are produced by mating between *L* and *R* or *L* and E^*^ (or E^**^), and because gametes of new hybrids do not carry exclusively the sexual chromosome X, as it is the case in old *P. esculentus E* (Berger 1988; Berger et al. 1988). The following equation is used to calculate the weighted number of breeding events resulting in new sterile hybrids E^*^:

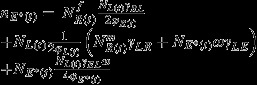
4

The same equation is used for new fertile hybrids (E^**^). The parameter *ω* represents the fitness of the new hybrids (Quilodrán et al. 2014), in which zero represents sterile individuals, whereas a value of one corresponds to individuals as fertile as the old *P. esculentus E*. We assumed that the old and the new hybrids, the fertile and the sterile ones, have the same survival parameters and mating preferences.

### The exotic frogs

Breeding events producing males and females *P. ridibundus* (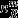, 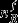) are also calculated separately due to the presence of crosses with old hybrids *E*, resulting in a sex bias in the offspring population in favour of females. Male and female populations are thus given by eqns 5 and 6, respectively.

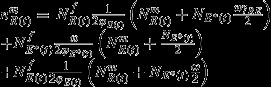
5

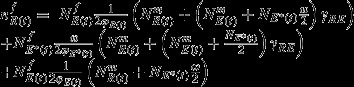
6

We used similar demographic and interbreeding success parameter values than those previously published in studies of the *L*/*E* system to find comparable equilibrium states (Hellriegel and Reyer 2000; Som et al. 2000, see Appendix S2). Then, we introduced *P. ridibundus* into the system. *P. esculentus* was considered to have a panmictic reproduction with *P. lessonae* (*γ*_*EL*_ = 1), while *P. lessonae* was assumed to have 10% of interbreeding success rate with *P. esculentus* (*γ*_*LE*_ = 0.1). We considered the same panmictic reproductive behaviour for *P. esculentus* relative to *P. ridibundus* (*γ*_*ER*_ = 1). We explored different levels of interbreeding success rate of the invasive *P. ridibundus* in relation to both native *P. esculentus* (*γ*_*RE*_) and *P. lessonae* (*γ*_*RL*_).

As in eqn 4 of Quilodrán et al. (2014), the temporal dynamics of adult populations is evaluated by adapting a version of the Ricker model, considering the intra- and interspecific density-dependent competition during a larval stage (Wilbur 1976). As incorporated by Henson et al. (2001), we considered the ‘lattice effects’, in which the simulated population dynamics could be different from the expected one due to the discrete nature of individuals in a population. This effect is introduced in our model through the rounding off operation in eqn 7:

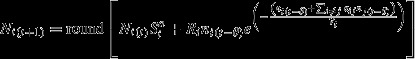
7

where *i* and *j* denote a population of *L*,E, E^*^, E^**^ or *R*. The first term in eqn 7 characterizes the abundance of adults surviving from the last to the current breeding period, for which 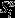 is the adult survival probability. The second term represents the surviving progeny until sexual maturity, where *θ* specifies the time to reach adult age in *t *+* *1. The value *R*_*i*_ indicates the number of hatchlings per breeding pair surviving until maturity (population growth rate). The parameter *α*_*ij*_ represents the interspecific competition between *i* and *j*. This parameter can take values ranging from 0 (no competition) to 1, in which case an individual from population *j* competes with an individual from population *i* as if they were members of the same population *i*. *V*_*i*_ denotes the habitat size, where 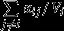 delimits the interspecific density-dependent mortality before the sexual maturity stage (Henson et al. 2001).

In nature, the abundance of the pre-existing hybrid *P. esculentus* relative to the population size of *P. lessonae* varies from 10% to 90% (Tietje and Reyer 2004). These variations may be related to habitat characteristics. Small ponds with abundant vegetation are favourable to *P. lessonae*;*P. ridibundus* prefers larger ponds with little vegetation and high levels of dissolved oxygen whereas habitats for *P. esculentus* are intermediate between *P. lessonae* and *P. ridibundus* (Holenweg Peter et al. 2002). We simulated several conditions of habitat size available for *P. lessonae* relative to the habitat size available for the exotic *P. ridibundus*: equal size (*V*_*L*_ = *V*_*R*_), 50% greater for *P. lessonae* (*V*_*L*_ > *V*_*R*_) or 50% smaller for *P. lessonae* (*V*_*L*_ < *V*_*R*_). As hybrid's habitat preference is intermediate between the ones of parental species (Holenweg Peter 2001), we assumed an intermediate habitat size (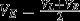) and an intermediate competition between hybrids and parental species (*α*_*EL*_ = *α*_*LE*_ = *α*_*ER*_ = *α*_*RE*_ = 0.5). As *P. ridibundus* and *P. lessonae* live in different habitats, no interspecific competition was assumed between them (*α*_*RL*_ = *α*_*LR*_ = 0) (but see Appendix S3 for an exploration of interspecific competition between both species).

The different abundance registered between both native frogs in the *L*/*E* system may also be motivated by different fertilities. The clutch size of *P. lessonae* is usually smaller than that of the hybrids *P. esculentus* (Tietje and Reyer 2004), both of which are also smaller than the clutch size of *P. ridibundus* (Ivanova and Zhigalski 2011). To take into account the fertility and the survival rate until maturity, we compute the parameter *R*_*i*_ in eqn 7 as the product of the clutch size (*c*_*i*_) and the survival probability across all stages until the age of sexual maturity (*b*), 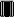 as follows:

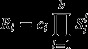
8

To calculate the population growth rate parameter, we considered the survival at tadpoles and metamorphs (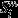) and the survival of first and second year juveniles (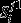, 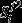). As the clutch size is strongly correlated with female body size (Schmeller et al. 2007) and knowing that hybrids exhibit intermediate body size between both parental species (Rist et al. 1997), we considered *P. esculentus* females to lay 30% more eggs than *P. lessonae* and 30% less eggs than *P. ridibundus* (*R*_*L*_ < *R*_*E*_ < *R*_*R*_). However, the higher survival of *P. lessonae* tadpoles may reduce (or counterbalance) the initial outnumber of hybrid and *P. ridibundus* tadpoles (Semlitsch and Reyer 1992). Thus, we also simulated ponds equally productive for all waterfrogs (*R*_*L*_ = *R*_*E*_ = *R*_*R*_). The survival of juveniles and adults seems to be similar between *P. lessonae* and *P. esculentus* (Tietje and Reyer 2004). In the absence of empirical data, we considered these values to be equal among the three waterfrogs (Table1). Values of fecundity and survival were obtained from previous studies (Berven 1990; Hellriegel and Reyer 2000; Som et al. 2000; Tietje and Reyer 2004; Mayer et al. 2013).

**Table 1:** List of functions and parameters of the model with default values

Symbol	Definition
*N*_*i*_	Number of adult individuals of genotypic class *i* Initial size: *N*_*L*_ = *N*_*E*_ = 50; *N*_E^*^_ = 0; *N*_E^*^^*^_ = 0; *N*_*R*_ = 0
*n*_*i*_	Weighted number of matings leading to offspring of class *i*
*R*_*i*_	Population growth rate
*θ*	Time delay from hatching to age maturity in *t *+* *1 *θ*_*L*_ = *θ*_*E*_ = *θ*_*R*_ = 1 (2 years)
*c*	Clutch size *c*_*L*_ = 1250; *c*_*E*_ = 1250–1625; *c*_*R*_* *= 1250–2000
*S*^*m*^	Survival of metamorphs 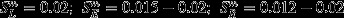
*S*^*j1*^, *S*^*j2*^	Survival of first and second year juveniles 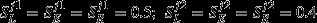
*S*^*a*^	Survival of adults 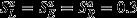
*α*	Interspecific competition coefficient *α*_*RL*_* *= *α*_*LR*_* *= 0; *α*_*RE*_ = *α*_*ER*_ = *α*_*LE*_* *= *α*_*EL*_ = 0.5
*V*	Habitat size *V*_*L*_ = 5000; *V*_*R*_ = 2500–5000
*γ*	Interbreeding success rate *γ*_*EL*_ = *γ*_*ER*_ = 1; *γ*_*LE*_ = 0.1; *γ*_*RE*_ = 0–1; *γ*_*RL*_ = 0–1; *γ*_*LR*_ = 0–1
*ω*	Fitness of newly formed hybrids 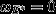 or 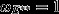

## Results

### The invasion of *P. ridibundus*

We assessed the consequences of the invasion of *P. ridibundus* coming from different geographical origins with differential capacities to induce hybridogenesis, on the native waterfrogs *P. lessonae* and *P. esculentus*. In the first 200 time steps (years), the populations of both native frogs reach stable equilibrium with different abundances, depending on the interbreeding success rate of *P. esculentus* (*γ*_*EL*_) and *P. lessonae* (*γ*_*LE*_), the productivity of each population (*R*_*E*_ and *R*_*L*_), and the habitat size available for each waterfrog (*V*_*E*_ and *V*_*L*_). The main results of the simulations in the *L*/*E* system indicated that: (i) the abundances of *P. lessonae* and *P. esculentus* observed in nature can be explained by different habitat size and/or unequal fertility between both frogs; (ii) the *L*/*E* system can persist with a panmictic reproduction of *P. esculentus* (*γ*_*EL*_ = 1) only if the interbreeding success rate is asymmetrical between both frogs with a smaller value for *P. lessonae* (*γ*_*LE*_ < 1); (iii) a random mate choice of *P. lessonae* (*γ*_*LE*_ = 1) always leads to the collapse of the system (see Appendix S2 for details).

**Figure 2:**
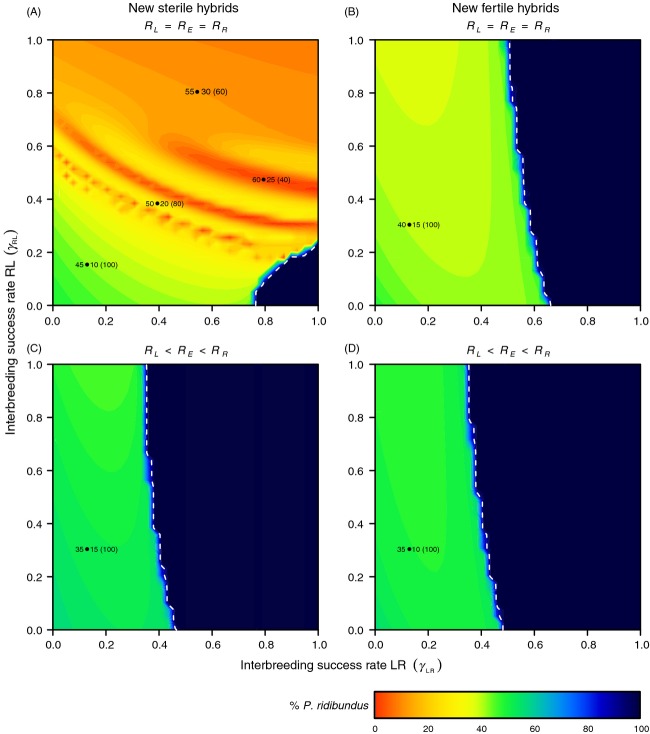
Effect of interbreeding success rate between *Pelophylax ridibundus* and *Pelophylax lessonae* on a waterfrog *L/E* system of Western Europe. (A, C) *P. ridibundus* introduced from Southern Europe, producing new sterile hybrids (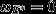). (B, D) *P. ridibundus* introduced from Central Europe, producing new fertile hybrids (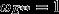). (A, B) Equally productive populations (*R*_*L*_ = *R*_*E*_ = *R*_*R*_); (C, D) *Pelophylax esculentus* has an intermediate fertility between *P. lessonae* and *P. ridibundus*, the latter being 60% more fertile than *P. lessonae* (*R*_*L*_ < *R*_*E*_ < *R*_*R*_). White dotted lines delimit the exclusive presence of *P. ridibundus* in the system. The colour scale represents the relative abundance of *P. ridibundus*, while the numbers inside the figure denotes the abundance of the other two frogs (%L•%E) and the percentage of replacement of old by newly formed hybrids, in brackets. We let the natives *P. lessonae* and *P. esculentus* interact during 200 time steps (years) before introducing *P. ridibundus*. Data correspond to the situation projected after a total of 400 time steps (years).

The equilibrium of this hybridogenetic system is disrupted when *P. ridibundus* is introduced (Fig.2). We explored an asymmetrical interbreeding success rate between *P. lessonae* and *P. ridibundus* (*γ*_*RL*_ ≠ *γ*_*LR*_), which could represent, for instance, a different mating preference between both taxa due to body size or mating vocalization. In this first analysis, the reproduction between *P. ridibundus* and *P. esculentus* is considered to be panmictic (*γ*_*RE*_ = 1), and all populations are assumed to be equally productive (*R*_*L*_ = *R*_*E*_ = *R*_*R*_).

Under this setting, when *P. ridibundus* comes from Southern Europe, producing new sterile hybrids (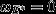), its frequency remains lower than 15% if its mating preference for *P. lessonae* is higher than 2:3 relative to the conspecific mating (*γ*_*RL*_ > 0.66). Some extreme situations are possible, in which the exotic frog can replace both native frogs, when the heterotypic preference of *P. lessonae* represents about 4:5 of the homotypic mating (*γ*_*LR*_ > 0.78). At this level of hybridization, native frogs can only remain if *γ*_*RL*_ > 0.23. Levels of interbreeding success rate lower than 40% for *P. ridibundus* (*γ*_*RL*_ > 0.4) and 60% for *P. lessonae* (*γ*_*LR*_ > 0.6) cause the extinction of old *P. esculentus* and the collapse of the hybridogenetic system. In this case, the system is composted mostly by *P. lessonae* and *P. ridibundus*, with a marginal population of new sterile hybrids (E^*^) that depends exclusively on the hybridization between both parental species (Fig.A).

When *P. ridibundus* comes from Central Europe, producing new fertile hybrids (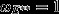), any level of hybridization skewed towards *P. ridibundus* or towards *P. lessonae* (*γ*_*RL*_ ≠ *γ*_*LR*_) allows the colonization of the exotic frogs in densities of at least 40% (Fig.B). The extinction of old *P. esculentus* due to the replacement by new fertile hybrids is always reached. If the proportion of heterotypic matings of *P. lessonae* represents 2:3 of the homotypic matings (*γ*_*LR*_ > 0.66), the extinction of all native frogs is reached and is almost independent from the interbreeding success rate of *P. ridibundus* (*γ*_*RL*_ ≠ 0). The invasion of the exotic frog is thus reinforced by the capacity of Central European *P. ridibundus* to produce fertile new hybrids.

When considering a higher number of individuals reaching sexual maturity in *P. ridibundus* and *P. esculentus* than in *P. lessonae* (*R*_*L*_ < *R*_*E*_ < *R*_*R*_), both geographical provenances of the invasive frogs (producing sterile or fertile hybrids) lead to similar results. *P*. *ridibundus* colonizes the area in frequencies of at least 50%, with old *P. esculentus* always replaced by newly formed hybrids, and with only *P. ridibundus* remaining if the interbreeding success rate of *P. lessonae* with *P. ridibundus* is higher than 0.5 (*γ*_*LR*_ > 0.5) (Fig.C,D). In such scenarios, a more productive population of *P. ridibundus* threatens both native frogs irrespective of the geographical origin or the capacity to induce hybridogenesis.

### The effect of available habitat size

**Figure 3:**
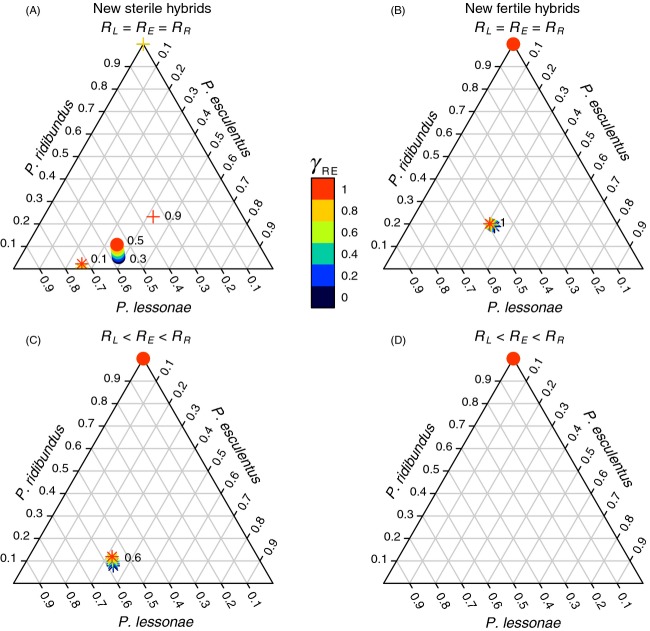
Estimated proportions of exotic *Pelophylax ridibundus*, native *Pelophylax lessonae* and *Pelophylax esculentus* in a *L*/*E* system of Western Europe. The grey lines inside the triplot indicate the relative abundance of each waterfrog. The horizontal line represents the abundance of *P. ridibundus*, while diagonals oriented to the left and the right represent the abundance of *P. lessonae* and *P. esculentus*, respectively. The colour scale represents the interbreeding success rate of *P. ridibundus* with *P. esculentus* (*γ*_*RE*_). The numbers located within the triplots are the proportion of newly formed *P. esculentus* (see text) within the total *P. esculentus* population (0 to 1). (A, C) *P. ridibundus* introduced from Southern Europe, producing new sterile hybrids (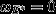). (B, D) *P. ridibundus* introduced from Central Europe, producing new fertile hybrids (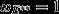). (A, B) Equally productive populations (*R*_*L*_ = *R*_*E*_ = *R*_*R*_); (C, D) *P. esculentus* has intermediate fertility between *P. lessonae* and *P. ridibundus*, the latter being 60% more fertile than *P. lessonae* (*R*_*L*_ < *R*_*E*_ < *R*_*R*_). (•) Equal habitat size between *P. lessonae* and *P. ridibundus* (*V*_*L*_ = *V*_*R*_). (+) Habitat size of *P. lessonae* 50% smaller than the one available for *P. ridibundus* (*V*_*L*_ < *V*_*R*_). (*) Habitat size of *P. lessonae* 50% greater than the one available for *P. ridibundus* (*V*_*L*_ > *V*_*R*_). We let the natives *P. lessonae* and *P. esculentus* interact during 200 time steps (years) before introducing *P. ridibundus*. Data correspond to the situation projected after a total of 400 time steps (years).

The effects of habitat size differences between *P. lessonae* and *P. ridibundus* on the *L/E* system, and the effects due to the interaction between native and exotic frogs were simulated for a period of 200 time steps (years) (Fig.3). In this analysis, we simulated different levels of submerged vegetation and dissolved oxygen favouring one or the other species (see Methods). The aim was to represent a variety of ponds in a given colonized region and to explore the effect of an increasing mating between the invasive frog and the hybrid *P. esculentus* (*γ*_*RE*_). We assumed an extreme case of symmetrical and panmictic interbreeding success rate between parental taxa (*γ*_*LR*_ = *γ*_*RL*_ = 1).

Taking into account equally productive populations (*R*_*L*_ = *R*_*E*_ = *R*_*R*_), when the habitat size of *P. ridibundus* is equal or smaller than the one available for *P. lessonae* (*V*_*L*_ = *V*_*R*_ or *V*_*L*_ > *V*_*R*_, Fig.3A), then the new sterile hybrids do not lead to the extinction of native frogs or to a complete replacement of old by newly formed hybrids. However, a greater habitat size available for *P. ridibundus* (*V*_*L*_ < *V*_*R*_) leads to the extinction of all native frogs, excepting the case in which the reproduction between *P. ridibundus* and the hybrid is panmictic (*γ*_*RE*_ = 1). In this case, the cost represented by the production of sterile hybrids (E^*^), which replace 90% of old hybrids *P. esculentus* and compete with the newly arrived frogs, restrict the invasion of *P. ridibundus*. A habitat size 55% greater for *P. ridibundus* as compared to *P. lessonae* leads to the extinction of all native frogs, irrespective of the level of interbreeding success rate with the hybrid *P. esculentus* (data not shown). For *P. ridibundus* coming from Central Europe and producing new fertile hybrids (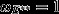), a complete extinction of both native frogs is expected if the habitat size of *P. lessonae* is equal or smaller than that available for *P. ridibundus* (*V*_*L*_ = *V*_*R*_ or *V*_*L*_ < *V*_*R*_, Fig.3B). A greater habitat size for *P. lessonae* (*V*_*L*_ > *V*_*R*_) allows the coexistence of all waterfrogs, but a complete replacement of old by new fertile hybrids (E^**^) is expected.

In the case of more productive populations of *P. ridibundus* and *P. esculentus* as compared to *P. lessonae* (*R*_*L*_ < *R*_*E*_ < *R*_*R*_), only an invasion of South European *P. ridibundus*, producing new sterile hybrids (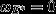), and having a smaller habitat size as compared to *P. lessonae,* allows the coexistence of all waterfrogs, but 60% of old *P. esculentus* is replaced by newly formed hybrids (Fig.3C). In contrast, a displacement of all populations occurs if *P. ridibundus* comes from Central Europe, producing new fertile hybrids (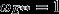), irrespective of the habitat size of the parental species and the interbreeding success rate between *P. ridibundus* and *P. esculentus* (Fig.3D). However, even in this extreme scenario of more productive populations of *P. ridibundus* (producing new fertile hybrids), a habitat size 2.1 times larger for *P. lessonae* allows the coexistence of all waterfrogs, but with a complete replacement of old by newly formed hybrids (data not shown). Otherwise, the system is composed exclusively of *P. ridibundus* (Fig.3D).

## Discussion

In the present study, we used the general model of interspecific hybridization without introgression that we developed recently (Quilodrán et al. 2014) to analyse and project possible future situations for the native *Pelophylax L*/*E* system in Western Europe. If we do not consider the recent introduction of *P. ridibundus* in this region*,* our model gives comparable results to previous studies which aimed at understanding the *L*/*E* system. In particular, we found equilibrium states that are equivalent to those reported by multistage life cycle models (Hellriegel and Reyer 2000; Som et al. 2000). Those results are congruent with some previous observations of waterfrog abundance reported in the *L*/*E* system (Semlitsch et al. 1997; Tietje and Reyer 2004). Moreover, the results obtained with our model are consistent with the suggestion of Holsbeek and Jooris (2010) who anticipated the negative impact of *P. ridibundus* on Western European waterfrogs.

### The invasion of *P. ridibundus*

The exotic *P. ridibundus* is currently replacing native frogs in Western Europe (Vorburger and Reyer 2003). However, individuals translocated from distinct geographical origins differ in their colonization capabilities and in the mode of invading the waterfrog community (Holsbeek and Jooris 2010; Plötner et al. 2010). The projections of our model suggest that *P. ridibundus* coming from Southern and from Central Europe are both a threat for the persistence of native waterfrogs in Western Europe.

When *P. ridibundus* is introduced from Southern Europe (producing new sterile hybrids), it threatens firstly old *P. esculentus* and also the persistence of the hybridogenetic system. But, the whole system of native waterfrogs can be threatened if the interbreeding success rate is skewed towards *P. ridibundus*, or when the invasive frog has a more productive population or a larger habitat size than native frogs. We also showed that *P. ridibundus* coming from Central Europe (producing new fertile hybrids) represents a direct threat for both native frogs in most situations. The production of unviable offspring resulting from homotypic matings in *P. esculentus* is critical for the persistence of the *L*/*E* system (Hellriegel and Reyer 2000; Som et al. 2000). With the arrival of *P. ridibundus* from Central Europe, this equilibrium is broken which eventually leads to the collapse of native frogs.

The area invaded by *P. ridibundus* in Western Europe is currently larger than was originally predicted (Schmeller et al. 2007). The first observation in Switzerland date back to 1950, and since then, this species has colonized the country with varying local success. Western Switzerland has been invaded more rapidly, and native frogs are at edge of extinction, while, in northern and eastern regions, all three taxa are still coexisting (Vorburger and Reyer 2003). We suggest that these two contrasting situations are due to the introduction of *P. ridibundus* coming from different geographical origins, displaying distinct capacities to induce hybridogenesis, as we show in Fig.2 (see *Conservation action* section below). In Germany, both the high abundance of *P. rudibundus* reported by Mayer et al. (2013) and the specific habitat loss of *P. lessonae* (Zahn 1996) may explain why *P. lessonae* is considered the most threatened frog of the system (Mayer et al. 2013), in agreement with our results in Fig.3.

Although some simulations support the persistence of the three taxa, especially when *P. ridibundus* comes from Southern Europe, producing new sterile hybrids, the population size of native taxa may be reduced. In such conditions, the census size might not be sufficient for the population to remain viable due to the interactions between demography and genetics that are not incorporated in our simulations. Inbreeding depression, for instance, leads to a loss of genetic diversity and a reduced capacity to adapt to environmental changes, which potentially produce extinction risk in front of demographic decline (Frankham et al. 2014).

### Conservation action

The results of our simulations show that knowing the provenance of *P. ridibundus* is central to situate the importance of the threat in a management programme. However, it is not possible to discriminate the origins of the exotic waterfrogs based on morphology. Nevertheless, it can be performed using bioacoustics measures (Schneider et al. 1993) or by the analysis of new genetic markers (e.g. Montoya-Burgos et al. 2010), or the mitochondrial 12s rRNA (Plötner 1998; Plötner and Ohst 2001) or the serum albumin intron 1 (SAI1) (Plotner et al. 2009). Crossing experiments is today the best way to recognize the reproductive properties characterizing the geographical origins, but they require infrastructure and are time consuming.

To design convenient conservation actions, our model opens the possibility to assess the effects of modifying some parameters of the system. It is thus possible to test and identify the best candidate conservation strategies. Our results indicate that an efficient action could be to increase the habitat size of native frogs relative to the favourable habitat of the exotic frogs. Ponds with submerged vegetation and with medium levels of dissolved oxygen are the preferred habitat of *P. lessonae*, which is also convenient for *P. esculentus*, while *P. ridibundus* performs poorly in such conditions (Holenweg Peter et al. 2002). In comparable situations, marsh habitat creation, restoration and management aiming at promoting population survival and growth of target species and improving the connectivity among populations have successfully increased the abundance of threatened amphibians (e.g. Rannap et al. 2009) while also indirectly reducing or eliminating invasive species in some cases (Adams and Pearl 2007). The identified conservation action directed towards favouring the native species rather than fighting the invasive one is all the more advantageous as controlling an invasive species, once settled, is extremely difficult and costly (Januchowski-Hartley et al. 2011). Moreover, the suggested action does not involve species identification in the field or in the laboratory, but takes advantage of the difference in the ecological niche between the invasive and native taxa.

Our results indicate that a worse-case scenario is when the relative productivity of *P. ridibundus* coming from Central Europe is higher than native waterfrogs, in which case enlarging the habitat size of *P. lessonae* is insufficient to allow the survival of native populations. Even though the clutch size of *P. ridibundus* is known to be greater than that of *P. lessonae*, basic information about the relative number of individuals reaching sexual maturity is still lacking, and this knowledge needs to be acquired based on observations in the field before we can assess the realism of this worse scenario. Nevertheless, if we assume that this case is real, using our model it is possible to determine the amount of *P. ridibundus* that need to be controlled to artificially reduce the productivity of *P. ridibundus* up to a level that allows the survival of native species under the condition of an enlarged habitat size for *P. lessonae*. As the species identification is not possible in the field, the control of *P. ridibundus* could be achieved by removing all waterfrogs or their clutch in a determined fraction of the most suitable habitat for *P. ridibundus*. In these ways, using the different ecological requirements of the species, it is possible to preferentially remove the invader. The development of habitat suitability index models have already been used to predict the expansion of invasive amphibians (Lobos et al. 2013) and may also help to reduce the cost of localizing individuals belonging to invasive species, improving eradication programmes (Hester and Cacho 2012). However, detailed knowledge about the differences in habitat suitability between the invasive and native taxa should be verified in the field before implementing any control programme.

### Hybrids in conservation policies

We explored the microevolutionary effects of interspecific hybridization without introgression and determined the conditions under which native species might be threatened. In general, hybrids do not fall into protection plans because they are not considered as independent evolutionary lineages (Kraus 1995). The latter statement may be true if hybrids are unviable or infertile, but not if hybrids generate self-propagating progenies through parthenogenesis, hybridogenesis or gynogenesis (Kraus 1995; Christiansen and Reyer 2009). Polyploid hybrids have been important in the evolution of several plant taxa and the role of hybridization in animal speciation cannot be neglected (Soltis and Soltis 1999; Choleva et al. 2012). Hybridization between *P. ridibundus* and *P. lessonae* is interesting not only because it is a well-studied case of hybridogenesis but also because populations of only hybrid frogs have been observed in nature (Christiansen and Reyer 2009), demonstrating the possible emergence of self-sustaining populations. Natural interspecific hybridization is thus a dynamic process with several possible evolutionary outcomes, and natural hybrids are at the centre of this process. This gives a conceptual ground explaining why the native hybrid *P. esculentus* should also be protected.

**Figure 4:**
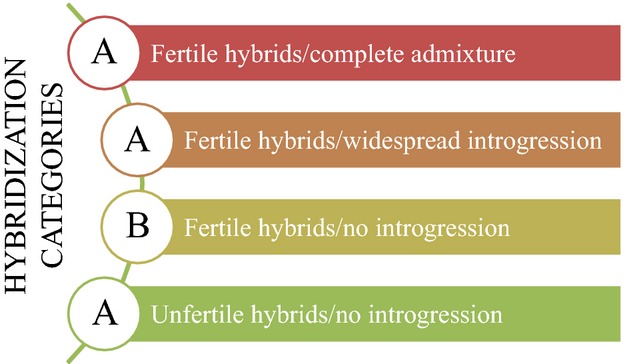
Hybridization categories. (A) classification proposed by Allendorf et al. (2001): (i) complete admixture, (ii) widespread introgression and (iii) hybrids without introgression due to infertile hybrids. (B) The new category we propose in this study, in which hybrids are fertile, but there is no genetic introgression between parental species because hybrids produce clonal or hemiclonal offspring.

To help prioritizing conservation actions, Allendorf et al. (2001) proposed three different categories of human-induced hybridization: (i) complete admixture, (ii) widespread introgression and (iii) hybrids without introgression due to infertile hybrids. In the first two categories, hybridization is a major threat to native species due to genetic introgression. The third category is believed to represent a minor risk because only F_1_ individuals are detected in nature, and thus, they are generally considered to be infertile, an assumption that can be wrong in an unappreciated proportion (Quilodrán et al. 2014). Here, we proposed a fourth category in which only F_1_ hybrids can be observed into the wild but producing a viable and fertile progeny that may dramatically impact the demography of one or both parental species (Fig.4). These hybrids do not undergo genetic recombination during their gametogenesis as they exclude one or the other parental genome in their germ cells, producing clonal or hemiclonal offspring. Therefore, this kind of hybrids does not mediate nuclear DNA introgression between parental species. As demonstrated here, this type of hybridization could represent a major risk by eventually replacing species via demographic means. In nature, several examples of this phenomenon have been documented, such as in the fish genera *Squalius* (Crespo-Lopez et al. 2006) and *Phoxinus* (Mee and Taylor 2012) and in the insects of genera Bacillus (Mantovani and Scali 1992). Moreover, Yakovlev et al. (2000) proposed that it could be the typical situation when distantly related fishes hybridize, which can be frequently seen in some families like the Cyprinidae (e.g. Schmidt et al. 2011). Therefore, the occurrence of this type of interspecific hybridization in other organisms is probably highly underappreciated, hiding a potentially important threat to species. We recommend that, in surveys or studies where only F_1_ individuals have been detected in nature, the genetic content of the hybrids' gametes should be verified to identify the risk that this interspecific hybridization may convey.

In addition to the conservation considerations discussed above, we believe that our model is a powerful tool for the study of a wide range of theoretical and empirical cases. The code of our model is freely available at: http://genev.unige.ch/montoya-currat/scripts/ and can be easily modified and adapted to investigate a variety of biological issues in conservation and evolution. Regarding the present case study on waterfrogs, further modifications of the model can potentially be incorporated when new information will be available. For example, possible differences in the survival parameters and mating preferences of new and old hybrids *P. esculentus* may be integrated, which were considered to be equal in our simulations. In a more general way, the model can be useful to understand the potential impact of unviable or sterile hybridization due to postzygotic barriers or to assess how different polyploid forms of hybrid origin can persist over large periods of time.

## Data archiving statement

The code for this study is available at: http://genev.unige.ch/montoya-currat/scripts/.

## Supporting Information

**Appendix S1.** A general model of nonintrogressive hybridization. **Appendix S2.** The hybridogenetic system of *Pelophylax lessonae* and *P. esculentus* (*L*/*E* system). **Appendix S3.** Introducing competition among parental species.
