# Supplementary material for: Modelling interspecific hybridization with genome exclusion to identify conservation actions: the case of native and invasive Pelophylax waterfrogs
Source: Evol Appl. 2015 Jan 28;8(2):199–210. doi: 10.1111/eva.12245 (PMC4319866; doi:10.1111/eva.12245)
Supplement: Supplementary file 1 [file eva0008-0199-sd1.pdf]

Supporting Information

Modeling interspecific hybridization with genome exclusion to identify conservation actions: the case of native and invasive *Pelophylax* waterfrogs

Appendix S1. A general model of non-introgressive hybridization

To explore the potential effect of the introduction of *P. ridibundus* and the hybridization with native frogs of Western Europe, we adapted a general model developed recently to study interspecific hybridization with unfertile hybrids or fertile hybrids without chromosomal recombination during gametogenesis (Quilodrán et al., In press). We briefly present the general model with a special emphasis on the formulas and the variables that have been adjusted to the present study of Western Europe waterfrogs.

The model considers diploid organisms, with parental genotypes codified as 00 and 11 for species N_0 and N_1, respectively. First-generation hybrids have genotype 01 and are noted as $N_{\frac{1}{2}}$. A genotype of class i has a probability M_{ij} to mate with individual of genotype class j, for all $i,j \in [0,...,1]$, as follow:

$$M_{i,j} = \frac{\gamma_{i,j} N_{j(t)}}{\phi_{i(t)}} \tag{1}$$

The parameter $\gamma_{i,j}$ represents the interbreeding success rate between i and j, which could be interpreted as a measure of assortative mating for the resulting values of $1-\gamma_{i,j}$. The value of $\phi_{i(t)}$ is a normalization factor such that $\Sigma_i M_{i,j} = 1$. Subsequently, we estimate the number of breeding pairs between i and j producing offspring k, weighted by the gametes fraction yielding k and the relative fitness of class k, as follow:

$$b_{ij,k(t)} = N_{i(t)} M_{ij(t)} C_{ij,k} \omega_k \tag{2}$$

where $C_{ij,k}$ is the relative fraction of offspring k produced by i x j. The value of ω_k represents the resulting fitness of an inherited character in the offspring k relative to parental species i and j. Therefore, the final weighted number of crosses producing k offsprings is estimated as the sum of all mating pairs producing individuals of class k

$$n_{k(t)} = \sum_i \sum_j b_{ij,k(t)} \tag{3}$$

The number of individuals reaching sexual maturity of each genotype class is computed with equation (7) presented in the main text. Details about the general model are given in Quilodrán et al. (In press).

Appendix S2. The hybridogenetic system of *Pelophylax lessonae* and *P. esculentus* (*L/E* system)

The *L/E* system is composed of *P. lessonae* (*L*) and the hybridogenetic hybrid *P. esculentus* (*E*). To assess the equilibrium states of the *L/E* system, and to evaluate if our model leads to analogous outcomes than those found in previous studies, we simulated scenarios with different habitat size (V_L and V_E), different population productivity (R_L and R_E), and different interbreeding success rate (γ_{LE} and γ_{EL}). Simulating different habitat sizes is justified because both waterfrogs use different habitats (Holenweg Peter et al., 2002). Simulating different fertilities is grounded on the fact that *P. esculentus* is known to have bigger clutch size than *P. lessonae* (Tietje and Reyer, 2004), although the higher survival rate at tadpole and metamorphic stages in *P. lessonae* may re-equilibrate the final number of individuals reaching sexual maturity in both waterfrogs (Semlitsch and Reyer, 1992). As to variations in the interbreeding success rate, we explored several scenarios in addition to those analyzed in previous studies.

The local abundances of *P. lessonae* (*L*) and *P. esculentus* (*E*) are extremely variable in nature (Tietje and Reyer, 2004). When we simulate $\pm 25\%$ of habitat size differences for each species, population sizes varies from 1:2 to 2:1 for *L*:*E* (Fig. S1), indicating that the variable abundance observed in nature may be explained by local dissimilar habitat sizes for each waterfrog. Simulations with higher habitat size dissimilarities increase the difference in local abundance between both frogs (data not shown).

The presence of *P. esculentus* in the frog community is highly dependent on the interbreeding success rates between both waterfrogs (γ_{LE} and γ_{EL}). For instance, with a null interbreeding success rate of *P. lessonae* ($\gamma_{LE} = 0$), where its females always mate with its homotypic males, females *P. esculentus* require levels of hybridization of 23% to 40% ($\gamma_{EL} = 0.23 - 0.4$) to persist in the community in every situations (Fig. S1b to S1f), except when the habitat size of *P. lessonae* is greater than that of *P. esculentus* ($V_L > V_E$) and both frogs are equally productive ($R_L = R_E$). In that later case *P. esculentus* always disappears if the heterotypic mating of *P. lessonae* (γ_{LE}) is close to 0, surviving only if $\gamma_{LE} > 0.05$ and the mate choice of *P. esculentus* is panmictic ($\gamma_{EL} = 1$) (Fig. S1a). When the heterotypic mating of *P. lessonae* is greater than 50% ($\gamma_{LE} \geq 0.5$) and that of *P. esculentus* is panmictic ($\gamma_{EL} = 1$), the system collapses in any case, with both frogs reaching extinction (Fig. S1).

Our results are comparable to the trends obtained in previous modeling studies of the *L/E* system, namely: 1) the various abundances of *P. lessonae* and *P. esculentus* observed in nature may be explained by different habitat size and/or unequal fertility between both frogs; 2) the *L/E* system can persist with a panmictic mate choice of *P. esculentus* ($\gamma_{EL} = 1$) only if the mate choice relaxation is asymmetrical between both frogs, smaller for P. lessonae ($\gamma_{LE} < 1$); 3) a panmictic mate choice of P. lessonae ($\gamma_{LE} = 1$) always leads to the collapse of the system (Graf, 1986; Hellriegel, 2000; Som et al., 2000). Therefore, with similar variables we get analogous outcomes, but our model allows us to study the effect of different parameters.

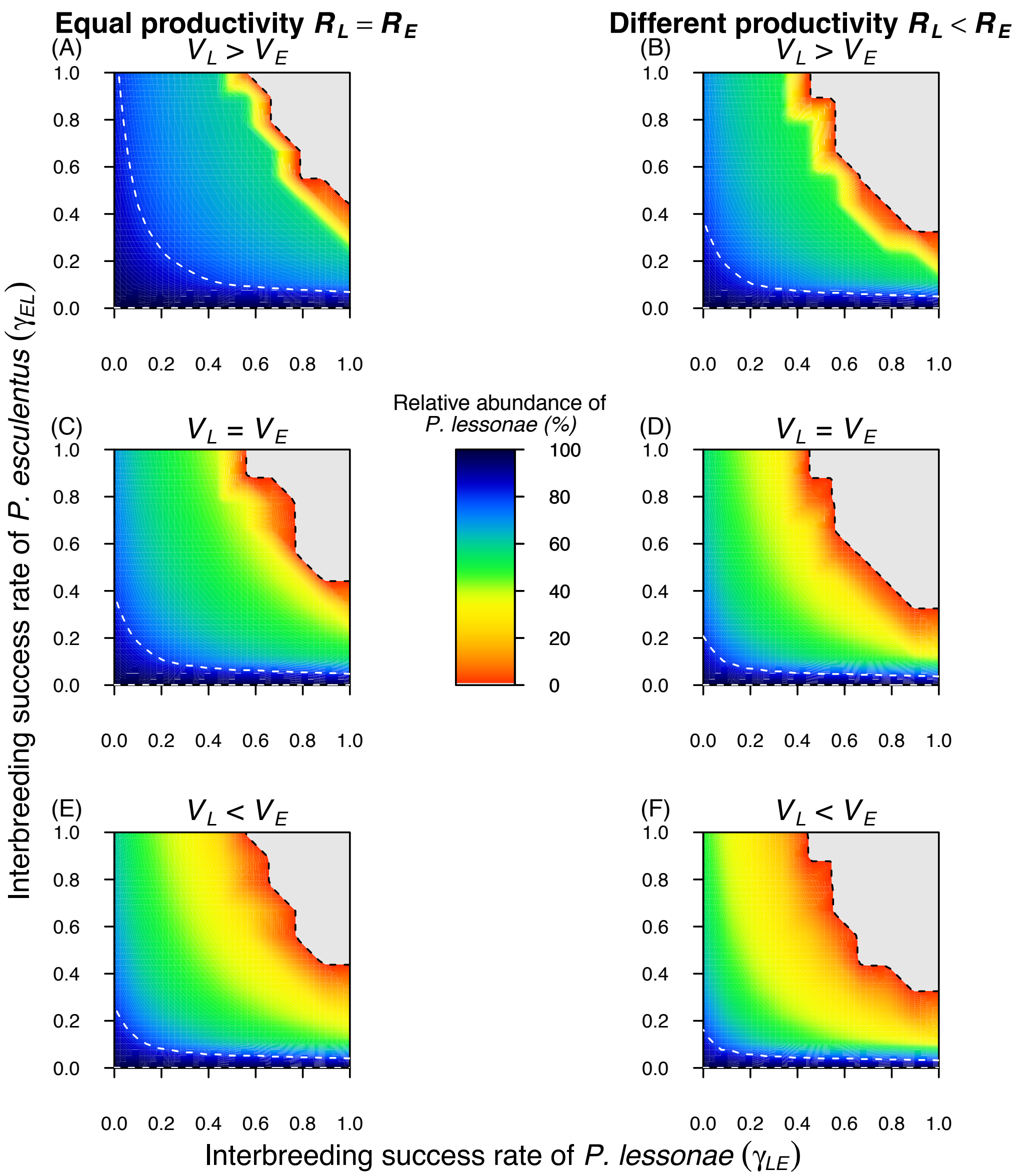

Figure S1. Relative abundance of *Pelophylax lessonae* as compared to *P. esculentus*. The dotted white lines delimit the exclusive presence of *P. lessonae*, while the dotted black lines delimit the collapse of the system, in which both native waterfrogs reach extinction. We simulated equally fertile populations ($R_L = R_E$) and also *P. esculentus* 1.3 times more fertile than *P. lessonae* ($R_L < R_E$). We took into account $\pm 25\%$ of different habitat size for both waterfrogs ($V_L < V_E$ or $V_L > V_E$). Parameter values were obtained from the literature (see Table 1, main text). Data are presented after 200 time-steps (years).

Introducing *P. ridibundus* into the *L/E* system

To generate the starting condition of the *L/E* system before the introduction of *P. ridibundus*, we used the conditions most often found in nature, which also correspond to the conditions

implemented in previous studies (Graf, 1986; Hellriegel, 2000; Som et al., 2000), i.e. a panmictic reproduction of *P. esculentus* ($\gamma_{EL} = 1$) and around 10% of heterotypic mating for *P. lessonae* ($\gamma_{LE} = 0.1$). Under these conditions and with an equal habitat size and fertility between both frogs, *P. lessonae* and *P. esculentus* persist, reaching a stable equilibrium at 60% and 40% of relative densities, respectively (Fig. S2, on the left side of the red line).

In the main text, we present various outcomes resulting from the introduction of *P. ridibundus* (*R*) into the *L/E* system obtained after 200 time-steps (years) following the translocation of the invasive waterfrog. In the present chapter, we describe the population dynamics of all waterfrogs during this time frame (Fig. S2, on the right side of the red bar). The outcomes depend on the geographic origin of *P. ridibundus* because Southern Europe individuals, when mating with *P. lessonae*, produce *P. esculentus* hybrids that are sterile in homotypic crosses, whereas Central Europe individuals produce *P. esculentus* hybrids that are fertile in homotypic crosses (see main text). Here, we consider equal habitat size ($V_L = V_E = V_R$) and equal productivity ($R_L = R_E = R_R$) among waterfrogs, with panmictic mate choices with *P. ridibundus* ($\gamma_{LR} = \gamma_{RL} = \gamma_{RE} = 1$). When *P. ridibundus* comes from Southern Europe, all waterfrogs persist in the community (Fig. S2a), with *P. lessonae* having the highest abundances, followed by the new formed hybrids and *P. ridibundus*. These final abundances can dramatically change depending on the parameter values, with, for example, a complete replacement of native frogs by *P. ridibundus* if the interbreeding success rate is highly asymmetrical ($\gamma_{LR} > \gamma_{RL}$) or if *P. ridibundus* and hybrids are considered to be more productive than *P. lessonae* ($R_L < R_E < R_R$) (see other scenarios in the main text). When *P. ridibundus* comes from Central Europe, both native frogs reach extinction (Fig. S2b). In this last scenario, the old *P. esculentus* (and the hybridogenetic system) disappears after 30 time-steps while *P. lessonae* reaches extinction after 40 time-steps. Since that moment, the community is composed exclusively of the invasive *P. ridibundus*.

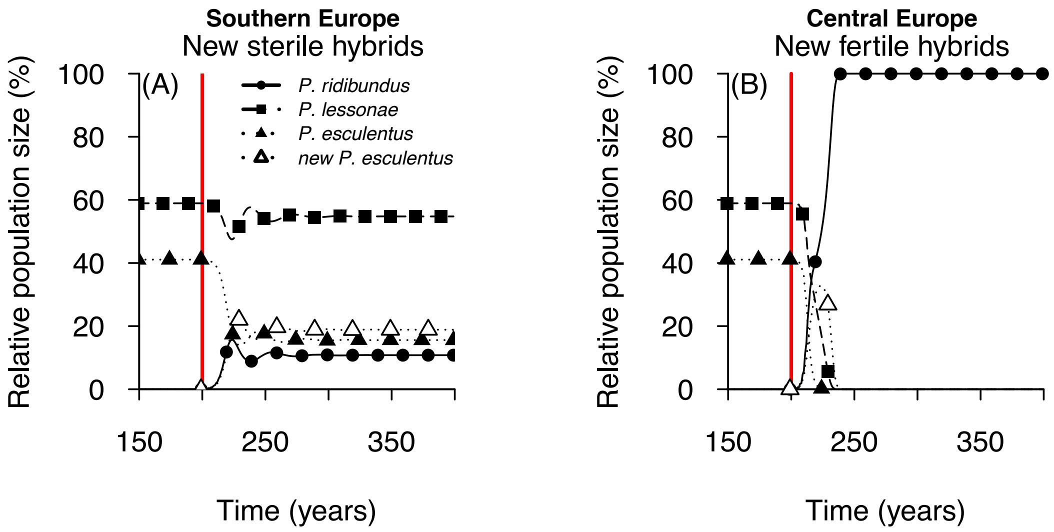

Figure S2. Relative population size of waterfrogs in a community of Western Europe. We let the natives *Pelophylax lessonae* and *P. esculentus* interact during 200 time steps (years) before introducing *P. ridibundus* (on the red line). a) New sterile hybrids; b) New fertile hybrids. We assumed panmictic reproduction ($\gamma_{LR} = \gamma_{RL} = \gamma_{RE} = 1$) and equally fertile populations ($R_L = R_E$). Other parameter values are presented in Table 1 (see main text).

Appendix S3. Introducing competition among parental species

In our analyses we included competition between the hybrid (*P. esculentus*) and the parental species *P. ridibundus* or *P. lessonae* but we did not incorporate interspecific competition between parental species as they use different habitats (Holenweg Peter et al., 2002). However, because both frogs can mate with each other, some competition may be expected, at least during the tadpole stage. We thus simulated also the effect of an increasing interspecific competition between *P. ridibundus* and *P. lessonae* ($\alpha_{RL} = \alpha_{LR} = \alpha$).

We first considered equally productive waterfrog populations (Fig. S3). When *P. ridibundus* comes from Southern Europe, producing new sterile hybrids, and with an interbreeding success rate between parental species of 10% ($\gamma_{RL} = 0.1$; Fig. S3a), *P. ridibundus* disappears if both parental species compete for more than 33% of the resources ($\alpha > 0.33$) and if it cannot mate with the hybrids ($\gamma_{RE} = 0$). If the intersepcific competition level is lower than 33% ($\alpha < 0.33$), the abundance of *P. ridibundus* increases with decreasing competition. However, if *P. ridibundus* is able to mate with the hybrids ($\gamma_{RE} > 0$), then the abundance of *P. ridibundus* decreases with increasing interspecific competition or tend to stabilize if its interbreeding success rate with the hybrid is greater than 40% ($\gamma_{RE} > 0.4$). When *P. ridibundus* comes from Central Europe, producing new fertile hybrids, its abundance is independent of the heterotypic mating with *P. esculentus* (γ_{RE}). The abundance of *P. ridibundus* remains ap-

proximately stable if it uses less than a half of the resources available for *P. lessonae* ($\alpha < 0.5$) whereas it displaces all native frogs if it uses more ($\alpha > 0.5$; Fig. S3a).

Similar trends are found when the hybridization between parental species is higher ($\gamma_{RL} = 0.5$; Fig. S3b). If the exotic frog comes from Southern Europe, less competition is required to reduce its abundance up to extinction, or, if they come from central Europe, less competition is required to displace the whole community of native frogs. In this last case, if *P. ridibundus* competes for more than 10% of the resources with P. lessonae ($\alpha > 0.1$), both native frogs disappear independently of the heterotypic mating between *P. ridibundus* and the hybrids *P. esculentus* (γ_{RE}) (Fig. S3b).

In summary, if we assume interspecific competition between both parental species (α), the abundance of Southern European *P. ridibundus* decreases with increasing competition. At the opposite, competition has a positive effect on the abundance of Central European *P. ridibundus*. With an increased interbreeding success rate between parental species (γ_{RL}), less competition is required for a complete replacement of native frogs by *P. ridibundus* if they come from Central Europe. However, if they come from Southern Europe, an increased hybridization between parental species leads to a decrease of the relative abundance of *P. ridibundus*. The heterotypic mating with hybrids (γ_{RE}) has no effect on the abundance of Central European *P. ridibundus*, but has a positive effect if they come from Southern Europe.

We finally considered a more productive population of *P. ridibundus* and of hybrids than of *P. lessonae* ($R_L < R_E < R_R$), the abundance of *P. ridibundus* increases with increasing competition, up to the replacement of native frogs when competition is strong, and this trend is independent of the geographical origin of *P. ridibundus* and the interbreeding success rate with hybrids (data not shown).

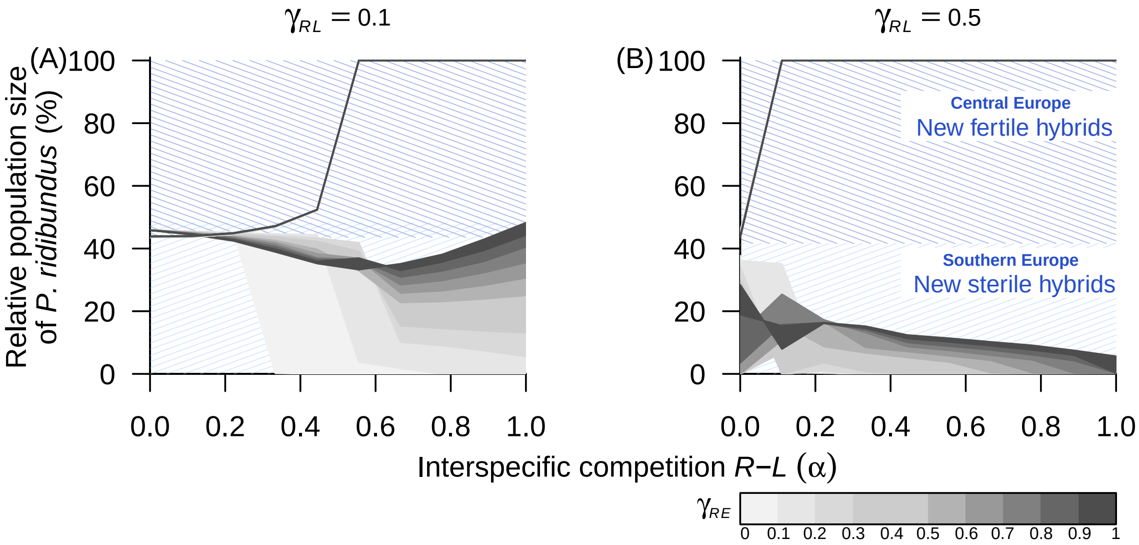

Figure S3. Effect of interspecific competition between *P. ridibundus* and *P. lessonae* in the colonization of the waterfrog community by *P. ridibundus* in Western Europe. a) Interbreeding success rate between parental species of 10%; b) Interbreeding success rate between parental species of 50%. We assumed symmetrical interbreeding success rate and competition between parental species ($\gamma_{RL} = \gamma_{LR}$ and $\alpha_{RL} = \alpha_{LR} = \alpha$). Data are presented after 400 time steps (years).

Literature Cited

Graf, J. D. 1986. Population genetics of the rana esculenta complex: a model. Studies in herpetology pages 175–180 .

Hellriegel, B. 2000. Single- or multistage regulation in complex life cycles: does it make a difference? Oikos **88:**239–249. ISI Document Delivery No.: 287QV Times Cited: 32 Cited Reference Count: 33 Hellriegel, B Munksgaard int publ ltd Copenhagen.

Holenweg Peter, A. K., H. U. Reyer, and G. A. Tietje. 2002. Species and sex ratio differences in mixed populations of hybridogenetic water frogs: The influence of pond features. Ecoscience **9:**1–11. ISI Document Delivery No.: 541GM Times Cited: 8 Cited Reference Count: 71 Peter, AKH Reyer, HU Tietje, GA Universite laval St foy.

Quilodrán, C. S., M. Currat, and J. I. Montoya-Burgos. In press. A general model of distant hybridization reveals the conditions for extinction in atlantic salmon and brown trout. Plos One .

Semlitsch, R. D., and H. U. Reyer. 1992. Performance of tadpoles from the hybridogenetic rana esculenta complex: interactions with pond drying and interspecific competition. Evolution **46:**665–676.

Som, C., B. R. Anholt, and H. U. Reyer. 2000. The effect of assortative mating on the coexistence of a hybridogenetic waterfrog and its sexual host. American Naturalist **156:**34–46. ISI Document Delivery No.: 326DZ Times Cited: 32 Cited Reference Count: 42 Som, C Anholt, BR Reyer, HU Univ chicago press Chicago.

Tietje, G. A., and H. U. Reyer. 2004. Larval development and recruitment of juveniles in a natural population of rana lessonae and rana esculenta. Copeia **3:**638–646. ISI Document Delivery No.: 849FL Times Cited: 6 Cited Reference Count: 45 Tietje, GA Reyer, HU Amer soc ichthyologists herpetologists Charleston.
